# Construction of physical crosslink-based chitosan/liquid crystal composite hydrogel and evaluation on their cytocompatibility

**DOI:** 10.1093/rb/rbw035

**Published:** 2016-10-26

**Authors:** Lin Du, Xiaohui Yang, Wenqiang Li, Xuhui Luo, Hao Wu, Jiaqing Zhang, Mei Tu

**Affiliations:** 1 Department of Materials Science and Engineering, Jinan University, Guangzhou 510632, People's Republic of China; 2 Engineering Research Center of Artificial Organs and Materials, Ministry of Education, Jinan University, Guangzhou 510632, People's Republic of China; 3 The First Affiliated Hospital, Jinan University, Guangzhou 510632, People's Republic of China; 4 School of Medicine, Jinan University, Guangzhou 510632, People's Republic of China

**Keywords:** chitosan, liquid crystal, composite hydrogels, structure and morphology, cell compatibility

## Abstract

In order to provide a novel biomimetic composite substrate for tissue engineering and explore the interaction between cells and this type of material, we developed chitosan/liquid crystal (CS/LC) composite hydrogel with embedded LC phases by composing of cholesterol hydroxypropyl cellulose ester liquid crystalline material and CS. The micromorphology of CS/LC composite hydrogels exhibited ‘islands-sea’ phase separation structures similar to the ‘fluid mosaic model’ of biomembrane. In vitro cell compatibility study suggested that 3T3 is fibroblasts exhibited better initial cell adhesions and higher proliferation rates on the composite hydrogel than on the polystyrene control plate and the pure LC membrane. This novel CS/LC composite hydrogel provides more favorable interface for cell growth and proliferation and may serve as potentially active substrate for engineering interfaces to live cells.

## Introduction

The interest in hydrogels for tissue engineering applications has increased owing to their capacity of designable mechanical properties and water content. Hydrogels are water-swollen, crosslinked polymer networks with mechanical behaviours similar to rubber-like materials [1–3], such as tissue-like elasticity and time-dependent viscoelastic behaviours. Therefore, they are flexible and possess similar properties as soft tissues, cartilage, skin or blood vessels, which make them highly suitable for the use in human body. Naturally derived hydrogels include crosslinked proteins, polypeptides and polysaccharides, in which chitosan (CS), collagen and alginate have been investigated as a matrix to support and promote the regeneration of new tissues in tissue engineering [4–7].

Chitin and CS belong to the family of glycosaminoglycans and constitute a series of the linear copolymers of N-acetyl-D-glucosamine and D-glucosamine with β (1 → 4) glycosidic linkages [8]. They have been widely used in cells encapsulation, cell culture [9], bone reconstruction and pharmaceutical fields for their bioactivity, biocompatibility, biodegradability and interesting physicochemical properties [10]. Mizuno *et al.* [11] reported that CS is able to incorporate basic fibroblast growth factors to accelerate the rate of wound healing. Park *et al*. [12] also studied the *in vitro* effect of carboxymethyl CS on the proliferation of normal human skin and keloid fibroblasts, and their results showed that CS accelerated the proliferation of keratinocytes.

Recently, design of biomimetic materials that are able to interact with surrounding tissues by biomolecular recognition has been widely researched [13, 14]. Particular attention has been paid to the design of biomimetic substrate for tissue engineering applications. Construction of CS /liquid crystal (LC) composite hydrogel will be a novel strategy based on biomimetic idea for generating an excellent artificial extracellular matrix (ECM) to create sites for cell recognition and specific physiological response [15]. Actually, LC science has been getting greater consideration in biosciences, and liquid crystalline materials that mimic biological environment and system have been applied for biomedical applications increasingly [16, 17]. The liquid-crystalline state widely exists in biological systems, e.g. the cell membranes and concentrated solutions of biomolecules such as DNA and proteins, are LCs. Liquid crystalline polymers are compatible with living systems and useful in the field of bioegineering due to their self-organized structure via noncovalent specific interactions [18–20]. Cholesterol liquid crystalline materials have especially raised more and more attention and interest in the field of biomaterials. Hwang *et al*. [21] found cholesteryl-(L-lactic acid) showing consistent spatial orientations that result in the promotion of initial cell adhesion and the subsequent repeated delivery of vital biological molecules to cells at the scaffold interface. Nagahama *et al.* [22] synthesized cholesterol side-functionalized poly(depsipeptide-co-DL-lactide) as a biodegradable material, in which the cholesterol LC phases served as physical crosslinking points to form noncovalent network structures among the polymer chains. This biodegradable LC material would become a new class of implantable biomaterial for organs such as blood vessels and heart.

We previously prepared two types of polyurethane/hydroxypropyl cellulose ester (cholesteryl LC compound) composite membranes and studied their cell compatibility. The results indicated that the addition of hydroxypropyl cellulose ester to the polymer substrate led to phase separation and formation of LC domains on the membrane surface. The type of LC influenced the crystalline behaviour of the substrate, and both the concentration and type of LC would exert effects on the morphological features and cytocompatibility of composite membrane [23].

In this study, physical hydrogel of CS [24] was chosen as a matrix and composited with hydroxypropyl cellulose ester LC compound to constitute the CS/LC composite hydrogel having a liquid crystalline phase. The surface morphology and phase separation between LC and CS were characterized by X-ray diffraction (XRD), scanning electron microscopy (SEM) and atomic force microscopy (AFM). The cytocompatibility test was performed to study the interaction between cells and CS/LC composite hydrogel substrate and elucidate the effects of the liquid-crystalline phase structure on cytocompatibility. The present study aims to provide convincing experimental data for the better design of biomimetic hydrogel served as potential candidates for engineering biointerfaces.

## Materials and methods

### Chemicals and materials

CS (Mw = 350 000; Sigma, USA) was purified before use. All other reagents and solvents used in our experiments were of analytical grade. Octyl hydroxypropyl cellulose ester (OPC) [Mw = 93 700] and propyl hydroxypropyl cellulose ester (PPC) [Mw = 9310 °C, T_NI(PPC) _= 155.6°C were synthesized in our lab [25].

### Preparation of CS/LC composite hydrogel

#### Pre-measurement of CS

CS used in our experiments was purified by dissolving the sample at 2% (w/v) in a stoechiometric amount of aqueous acetic acid, then filtering after complete dissolution. The solution was precipitated by adding excessive amounts of ammonia followed by centrifugation. Prior to lyophilizing, the precipitate was copiously rinsed using distilled de-ionized water until a neutral pH was achieved. The degree of deacetylation (D.D) of CS determined by linear potentiometric titration [26] was 67.36%.

#### Crosslinking CS/LC composite hydrogel

CS was dispersed in HCl to conduct the amino protonation, after which 1, 2-propylene glycol was added with the same amount equal to HCl. A homogeneous 1 wt% CS solution was obtained after the solution was stirred for 1 day. Certain amount of hydroxypropyl cellulose ester LC (namely PPC and OPC) was dissolved in alcohol, which was then sprayed into the above CS solution under rapid stirring. We prepared four solutions with a mass ratio of CS to LC of 1:0.5, 1:1, 1:2 and 1:3, respectively. CS/LC alcogels were obtained by pouring the solutions into a mould followed by dehydration at 50°C. These CS/LC alcogels were then immersed in NaOH for 1 day with the concentration of 1 mol/l followed by a thoroughly wash using de-ionized water.

Our previous study [24] demonstrated that hydrogels obtained by solvent exchange with 1 mol/l NaOH presented a three-dimensional (3D) network microstructure full of nanopores, containing plenty of water in freezable states and possessing favorable mechanical strengths (compression modulus was 130 kPa). Moreover, NaOH with a concentration of 1 mol/l had no significant effect on the properties of hydroxypropyl cellulose ester. Hence, 1 M NaOH solution was a suitable choice to perform treatment for CS/LC composite hydrogel.

### Characterization of CS/LC composite hydrogel

#### XRD analysis

The XRD analysis of lyophilized CS/LC composite hydrogels with different LC contents was performed using an X-ray diffractometer (Rigaku Dmax-1200, Japan) with Cu K_α_ radiation at a generator voltage of 40 kV and a generator current of 20 mA. Samples were scanned within 2θ = 5°–60° at a scanning rate of 8° min ^−^ ^1^ and striding width of 0.01°.

#### SEM observations

CS/LC composite hydrogels with different contents of LC were lyophilized and then frozen in liquid nitrogen before slicing. The surface of the sliced samples was studied by SEM (PHILIPS, XL-30ESEM). Before SEM analysis, a thin gold layer was coated on the specimen surface using a sputter coater (BAL-TEC, SCD005) to prevent charge up.

#### AFM observation

The AFM observation of CS/LC (1:1(w/w)) composite hydrogel fixed in petri dish (Φ 3.5 cm) by agarose gel was performed using contact mode in phosphate buffer solution (PBS) by a Bioscope Catalyst Nanoscope-V (Veeco Instruments Inc., USA). A silicon nitride cantilever was used, which had a spring constant of 5 N/m and a resonance frequency of 150 kHz. The AFM images were processed with the Nanoscope software.

### Cell compatibility evaluation

#### Fibroblast cell culture and cell viability test

The activity of cultured cells was measured with MTT colorimetric assays. PPC and OPC membranes of 1 mm in thickness were fabricated. Thin films of CS hydrogel and CS/LC composite hydrogel were prepared. All the samples were transferred to 48-well plates, which were sterilized with ^60^Co irradiation. After the samples were rinsed with sterilized PBS, cells were seeded on each sample. Three parallel samples for each group were investigated.

Mouse embryonic fibroblasts 3T3 (provide by the Biomedical Engineering Laboratory of Jinan University) were used for the cell experiments. Cells were cultured in L-DMEM medium supplemented with 10% fetal bovine serum (FBS, Gibco, BRL) and 1% penicillin/streptomycin (Gibco, BRL) at 37°C in a 5% CO_2_ supplied incubator. At the designed period of 1, 3, 5 and 7 days, cells were further incubated for 4 h at 37°C after adding 100-μl 5 mg/mL MTT solution. The solution was removed, and 1 ml of lysis solution was added into each well to terminate the reaction. After another 4-h incubation with complete dissolution of the dark-blue crystal of MTT formazan, 200 μl of the clear solution was transferred to a 96-well culture plate. The absorbance of the content of each well was measured at a wavelength of 570 nm and the reference wavelength of 630 nm with a microplate reader on a spectrophotometer against a blank of lysis solution.

#### Cell morphology observation

Samples were prepared as described above, at the sample/cells co-culture periods of 3 and 6 days, the samples were rinsed gently three times with PBS, each time for a period of 10 min, and fixed with 2.5% glutaraldehyde in PBS for 30 min at 4°C. After dehydration in graded alcohols (75%, 85%, 95% and 100%), samples were mounted on copper stubs, coated with gold and examined by SEM with an acceleration voltage of 20 kV.

## Results

### XRD analysis of CS/LC composite hydrogels

**Figure 1.**
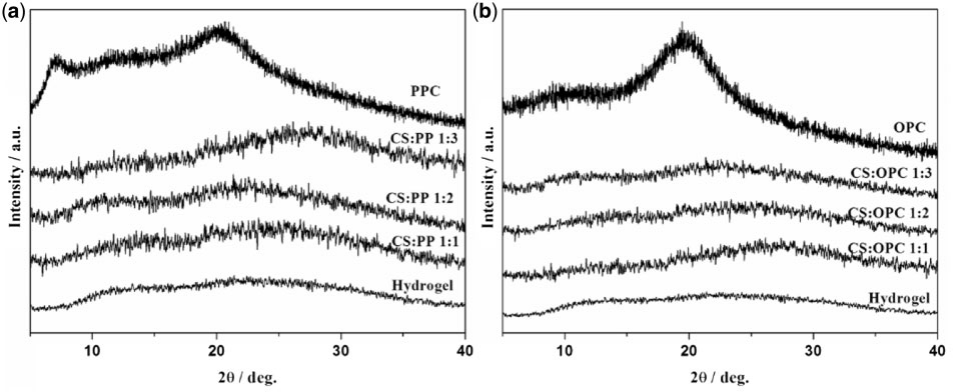
XRD of the CS/LC composite hydrogels.

Figure 1 shows the XRD patterns of the LC and CS/LC composite hydrogels. It is clear that the two Bragg peaks emerged at 2θ of 7° and 20° for PPC (Fig. 1a). However, they disappeared for the CS/PPC composite hydrogels with different LC contents, indicating that the CS/PPC composite hydrogels mainly presented the amorphous state in this case. The similar XRD pattern of CS/OPC was exhibited in Figure 1b, suggesting that CS/OPC composite hydrogels were in amorphous state as well, which were similar to that of pure CS hydrogel.

### SEM observations

**Figure 2.**
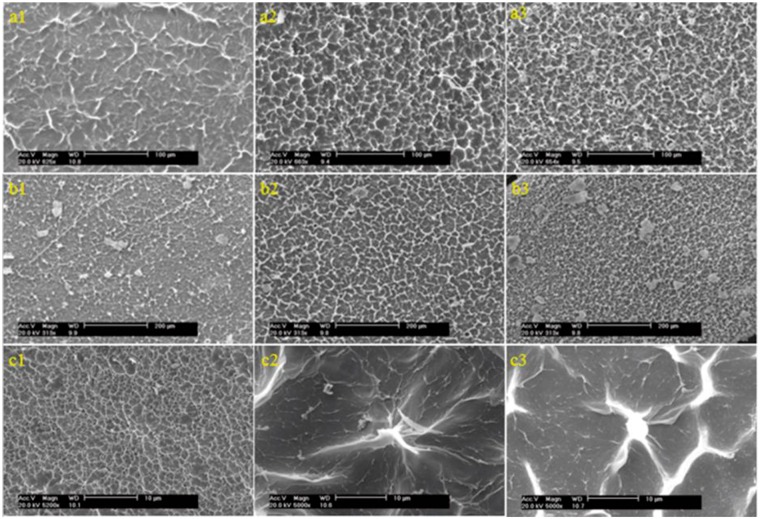
SEM images of CS/LC composite hydrogels. Shown in (**a1**)–(**a3**) and (**b1**)–(**b3**) are CS/PPC and CS/OPC composite hydrogels with a mass ratio of CS to LC 1:1, 1:2 and 1:3, respectively. Shown in (**c1**)–(**c3**) are CS hydrogel, CS/PPC with mass ratio of CS to PPC 1:0.5 and magnification of (a1), respectively.

The surface morphology of CS/PPC composite hydrogels was presented in Figure 2a1–a3. The SEM images showed that PPC phases dispersed uniformly on the CS matrix surface acting as physical crosslink points at a lower PPC content (Figure 2c2), but gathered gradually to form separate LC domain in the composite hydrogel with increased LC content (Figure 2c3). It seemed that the increase of LC content could cause phase separation between PPC and CS. The density of LC domains increased with PPC content. These domains began to connect with each other when the mass ratio of CS to PPC reached 1:1. However, the size of the LC domains did not change significantly in the range of 4–10 μm. Figure 2b1–b3 shows the surface morphology of CS/OPC composite hydrogels. OPC could disperse uniformly into the CS matrix, which is similar to CS/PPC as discussed above. With the increase of the LC content, the number of LC domains increased. We also observed that the domains connected into a stockwork structure with their sizes in the range of 4–10 µm.

**Figure 3.**
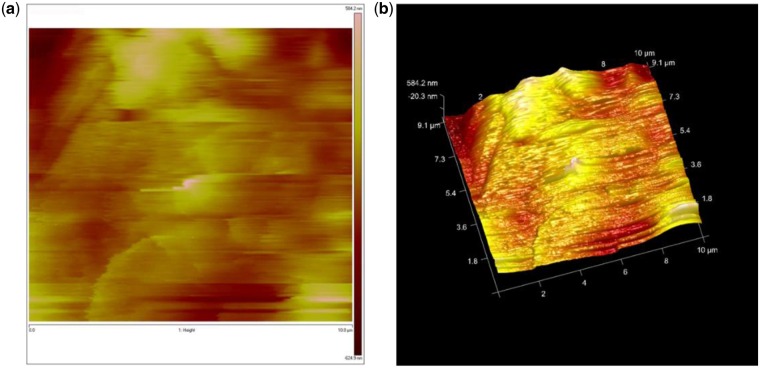
An AFM image (scan area: 10 µm ×10 µm) of a CS/PPC composite hydrogel (CS:PPC = 1:1(w/w)) presented in 2D (**a**) and 3D (**b**). The false color image has a height range of ∼1 µm, with the brighter color representing the taller features.

### AFM observations

In order to obtain more accurate LC state in the CS/LC composite hydrogel system, AFM was used in aqueous environment to study the LC morphology of a CS/PPC composite hydrogel with mass ratio of CS to LC 1:1. Figure 3a and b shows an AFM image presented in 2D and 3D, respectively. We believe that the lower features (orange in the false color AFM image) are the CS matrix and the higher features (yellow) the LC domains. More specifically, the LC domains embedded into the CS matrix and presented apparent bulges resembling islands. There were no clear boundaries between the CS matrix and the LC domains, rather, they connected together throughout the composite hydrogel. The size of the LC domains was in the range of 4–10 µm, which was consistent with the SEM observations. We also tried imaging a CS/OPC composite hydrogel, but could not obtain any reasonable AFM images because the sample was too soft and sticky.

### Cell compatibility of CS/LC composite hydrogels

**Figure 4.**
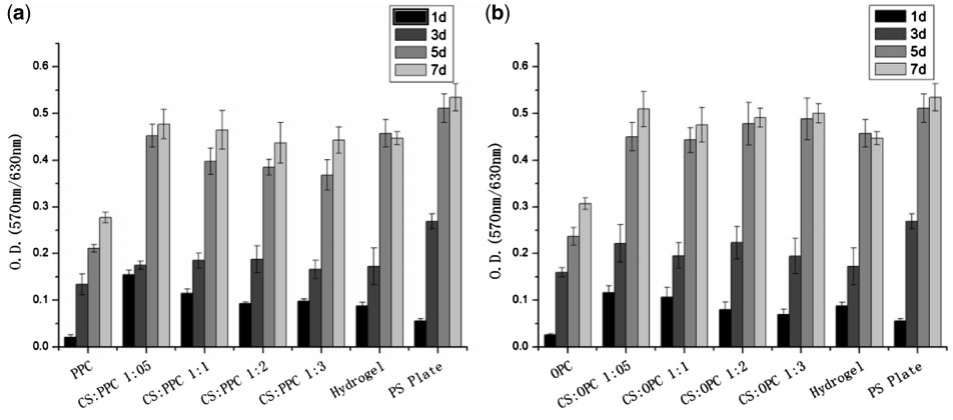
Values of optical density (OD) of cell cultivating *in vitro* on CS/LC composite hydrogel CS/PPC (**a**) and CS/OPC (**b**).

Figure 4 shows the viability of 3T3 fibroblast cultured on CS hydrogels, CS/PPC and CS/OPC composite hydrogels and pure LC membranes over different periods of 1, 3, 5 and 7 days, with the culture-grade polystyrene (PS) as the control. The results indicated that 3T3 fibroblast presented higher proliferation on the surface of CS/LC composite hydrogels than on that of the PS and the pure LC membrane at day 1 and then exhibited a logarithmic growth after contacting with all these materials for 3 days.

Generally, cells show high proliferation rate on the culture-grade PS during the culture time because PS is actually a good matrix for cell adhesion and growth. Although cell proliferation on the CS/LC composite hydrogels exhibited a little less than on the PS at later time point, cells still kept excellent viability and displayed higher proliferation rate at day 7. The main reason was attributed to the fact that LC domains embedded on the composite hydrogels provided the liquid-crystal–soft-solid interfaces that potentially favorable for cells growth and proliferation.

**Figure 5.**
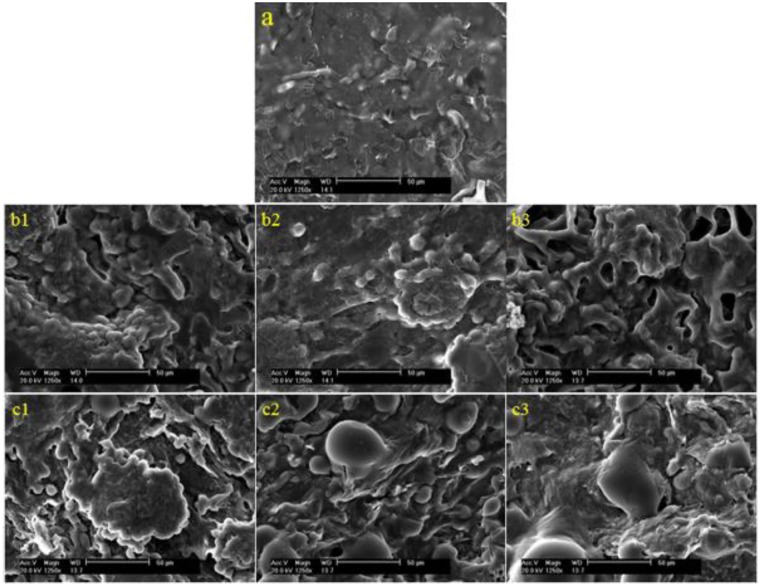
SEM images (×1250 magnification) of 3T3 cell cultivated *in vitro* on CS/LC composite hydrogels (3 days). Shown in (**a**) is CS hydrogel. Shown in (**b1**)–(**b3**) and (**c1**)–(**c3**) are CS/PPC and CS/OPC composite hydrogels with mass ratio of CS to LC 1:1, 1:2 and 1:3, respectively.

**Figure 6.**
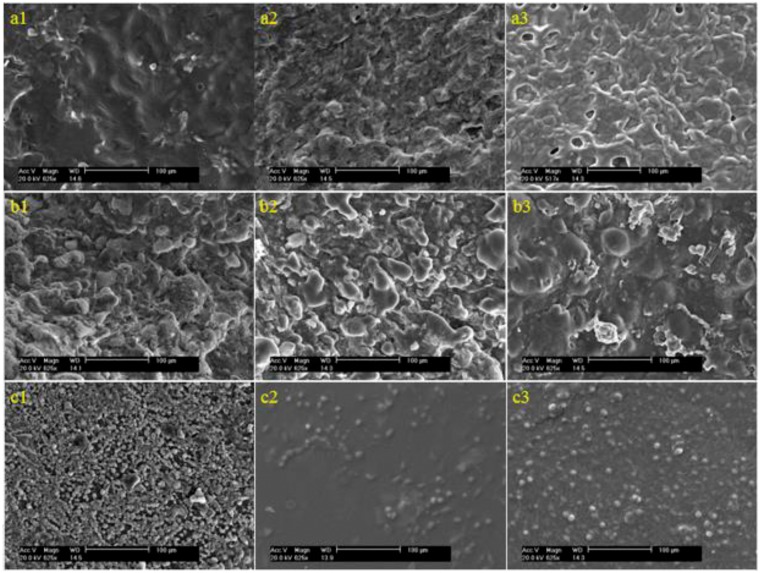
SEM images (×1250 magnification) of 3T3 cell cultivated *in vitro* on CS/LC composite hydrogels (6 days). Shown in (**a1**)–(**a3**) and (**b1**)–(**b3**) are CS/PPC and CS/OPC composite hydrogels with mass ratio of CS to LC 1:1, 1:2 and 1:3, respectively. Shown in (**c1**)–(**c3**) are CS hydrogel, PPC and OPC, respectively.

The cell morphology was studied using SEM after 3 days culture on the CS hydrogel and the CS/LC composite hydrogels with various mass ratio of CS to LC (Fig. 5). Cells exhibited global shape, a number of ECM formed around them and some cells connected each other via the ECM, indicating cells presented better viability on the substrate surface. Shown in Figure 6 are SEM images of 3T3 fibroblast after 6-day culture on a variety of materials. The substrate surface was covered with cells and ECM. Cells secreted more ECM on the composite hydrogels than on CS. Cells on the pure LC membrane just spread uniformly but did not reach out towards each other.

## Discussion

Previous work has demonstrated that many biological systems exist as ordered, liquid-crystalline materials [27]. In this study, two series of CS/LC composite hydrogels were prepared based on the biomimetic design guidance.

It was found that there were no distinct diffraction peaks in the XRD patterns of CS/PPC (OPC) composite hydrogels, indicating that LCs dispersed uniformly into CS instead of forming large block of self-aggregation due to the binding effect of 3D network structure of CS hydrogel, leading to an amorphous composite. The SEM images further indicated that the dispersed LC domains formed island-like structures in the matrix. Both LC and CS constituted the ‘fluid mosaic model’-like structure similar to that of biomembrane resulting in some particular reactions with cells.

Cell viability test and cell morphology observation were performed to explore the cytocompatibility of the CS/LC hydrogel. The results revealed that 3T3 fibroblast exhibited excellent viability on the CS/LC composite surface. The soft matter nature of hydrogel and the characteristic of LC combined in the composite might facilitate initial cell adhesion and remain prolonged cell viability. Especially, longer flexible side chain embedded in OPC endowed the CS/OPC composite with optimum viscoelasticity, which resembled closely the feature of soft matter cell model [28] and the deformable liquid-crystal–soft-solid interfaces [29] became potentially favourable for cells growth and proliferation. SEM observation of 3T3 fibroblast presented excellent cell growth and proliferation and a large number of ECM secretion on the CS/LC composite surface, which suggested that there may exist a synergy effect between CS and LC resulting in significant promotion to cell growth. It was well known that CS had a chemical structure similar to human body molecules-glycosaminoglycans [3] and CS hydrogel possessed the property of soft matter favoring cell attachment and growth. The addition of cholesteryl LC (PPC/OPC) into CS caused phase separation between the LC domains and the CS matrix, with the LC domains appearing as islands. This type of structure of CS/LC composite hydrogel might be explained by the ‘fluid mosaic model’ of biomembrane, offering consistent spatial orientation to promote initial cell adhesion and repeatedly providing active sites to cells at the substrate interface, which advanced cell proliferation and migration, as well as ECM secretion. The combination of the ordering and fluidity of soft matter nature in the composite hydrogel created the peculiar surface morphology that was likely to improve the interaction between CS/LC composite hydrogels and cells. Furthermore, blending of hydrophobic LC into hydrophilic CS might generate optimum hydrophilic–hydrophobic balance leading to a positive role for cell attachment and growth [30]. In addition, the mechanical strength of the composite hydrogel, especially the elasticity of this type of soft matter, might further offer a better condition for cell adhesion on the substrate surface. For the CS/LC composite hydrogel system, the ‘sensing’ LC domains continuously through CS matrix could act as active sites for the living cell and resulted in the higher degree of molecular mobility for binding interactions with cells and thus exhibited favourable cytocompatability [21].

## Conclusion

The CS/LC composite hydrogels investigated in this study could serve as active substrates for cells thanks to the combination of fluidity and ordered arrangement in this system, which had the similar movable morphology of the natural biomembrane surface and thus was compatible with living systems. Our results revealed that the CS/LC composite hydrogel system possessed biomimetic structures that made them potentially useful for engineering interfaces for live cells. It is proposed that the CS/LC composite hydrogel with the nature of soft elastic solid and a certain mechanical strength is a promising candidate for a new class of biomaterials.

## Funding

This work was supported by the Fundamental Research Funds for the Central Universities (21615436), the Science and Technology Program of Guangzhou, China (201508020035) and the Science and Technology Program of Guangdong, China (2016B090913004).

*Conflict of interest statement*. None declared.
